# The Need for Objective Quantitative Pigment Assessment in Comparative Laser Trials for Melasma

**DOI:** 10.1111/jocd.71112

**Published:** 2026-07-29

**Authors:** Tansha Anand, Neelshan Anand

**Affiliations:** ^1^ Peoples University of Medical and Health Sciences for Women Nawabshah Pakistan; ^2^ Bilawal Medical College LUMHS Jamshoro Pakistan

Dear Editor,

We read with great interest the recent randomized double‐blind split‐face trial by Wu et al., which compared 1064‐nm picosecond neodymium‐doped yttrium aluminum garnet (Nd:YAG) laser and 1064‐nm Q‐switched Nd:YAG laser for the treatment of melasma in Asian women. The authors should be commended for conducting a well‐designed prospective randomized study with blinded results assessment and a split‐face methodology, both of which strengthen the internal validity of the comparison. Their findings suggest that both laser modalities greatly improved melasma severity without statistically meaningful differences in efficacy, while picosecond treatment was linked with lower procedural pain.

Nevertheless, we would like to highlight a significant methodological consideration that may influence the interpretation of these outcomes. The study primarily evaluated treatment efficacy using the hemi‐modified Melasma Area and Severity Index (hemi‐mMASI) and the Global Aesthetic Improvement Scale (GAIS). Although these are widely accepted clinical outcome measures and the investigators improved reliability by employing two blinded dermatologists, both assessments remain largely dependent on clinical observation and subjective interpretation. As a result, subtle changes in pigmentation may not be appropriately assessed, particularly when comparing two treatment modalities that show relatively similar clinical performance.

Melasma is a complex pigmentary disorder characterized by heterogeneous epidermal, dermal, and mixed pigmentation. Clinical severity scores are significant for routine assessment; however, they cannot directly quantify melanin content or objectively differentiate small changes in pigment distribution. Modern non‐invasive imaging techniques, including reflectance spectrophotometry, colorimetric analysis, Mexameter measurements, standardized dermoscopy, and reflectance confocal microscopy, provide reliable quantitative information regarding pigment intensity and distribution that complements conventional clinical scoring systems. Such objective methods have increasingly been recommended in pigmentary disorder research because they decrease observer‐dependent heterogeneity and enhance the sensitivity for detecting treatment‐related changes, particularly in comparative clinical trials including laser therapies [1, 2].

This acknowledgment is particularly relevant in the present study because the observed variations between picosecond and Q‐switched Nd:YAG lasers were consistently numerical rather than statistically significant. In studies with relatively small sample sizes, minor improvements in pigmentation may remain undetected when result evaluation relies predominantly on subjective clinical scores. Incorporating objective pigment quantification alongside validated clinical indices could therefore enhance statistical precision and provide a more comprehensive assessment of treatment efficacy. Such multimodal evaluation may also enhance comparisons across studies and improve the reproducibility of future clinical trials [3, 4].

Furthermore, objective imaging may have particular relevance in monitoring disease recurrence. Melasma is well recognized as a chronic relapsing disorder, and microscopic pigment reaccumulation may precede clinically evident recurrence. Quantitative assessment techniques could therefore identify early pigment changes before they become visible on routine clinical examination, allowing a more accurate evaluation of long‐term treatment durability and maintenance strategies [2, 5].

Importantly, the authors themselves recognize that dermoscopic imaging was not systematically collected and recommend incorporating standardized objective imaging modalities in future investigations. We believe this represents a significant opportunity for future comparative laser studies. Integrating objective pigment assessment with validated clinical severity scores and patient‐reported results would strengthen the methodological rigor of melasma research and provide clinicians with stronger evidence when selecting between laser technologies.

In conclusion, we congratulate Wu et al. for their significant contribution to the growing evidence on laser therapy for melasma. Future randomized trials incorporating quantitative pigment measurement techniques in addition to conventional clinical scoring systems may further enhance the accuracy, reproducibility, and clinical applicability of comparative laser research.

## Author Contributions

Tansha Anand: Writing – original draft, conceptualization, writing – review and editing. Neelshan Anand: Writing – original draft, conceptualization, writing – review and editing.

## Funding

The authors have nothing to report.

## Ethics Statement

The authors have nothing to report.

## Conflicts of Interest

The authors declare no conflicts of interest.

## Data Availability

Data sharing not applicable to this article as no datasets were generated or analysed during the current study.
